# Cost-Effectiveness Analysis of Telehealth and In-Person Primary Care Visits for People Living with Alzheimer’s Disease-Related Disorders in the State of Nevada

**DOI:** 10.3390/ijerph21101381

**Published:** 2024-10-18

**Authors:** Yonsu Kim, Jay J. Shen, Ian Choe, Jerry Reeves, David Byun, Iulia Ioanitoaia-Chaudhry, Leora Frimer, Pengfeng Jin, Maryam Tabrizi, Hee-Taik Kang, Jae-Woo Lee, Claire Sieun Lee, Tae-Ha Chung, Yena Hwang, Ian Park, Hayden Leung, Jenna Park, Ji Won Yoo

**Affiliations:** 1 Department of Healthcare Administration and Healthcare Policy, School of Public Health, University of Nevada, Las Vegas, NV 89119, USA; yonsu.kim@unlv.edu (Y.K.); jay.shen@unlv.edu (J.J.S.); 2 Center for Health Disparities Research, School of Public Health, University of Nevada, Las Vegas, NV 89119, USA; 3 Digital Health Division, Nevada Optum Care, Las Vegas, NV 89128, USA; ian.choe@optum.com; 4 Department of Internal Medicine, Kirk Kerkorian School of Medicine at UNLV, Las Vegas, NV 89102, USA; david.byun@nv.touro.edu (D.B.); iulia.ioanitoaia-chaudhry@va.gov (I.I.-C.); leora.frimer@unlv.edu (L.F.); pengfeng.jin@unlv.edu (P.J.); claireunsi@gmail.com (C.S.L.); yenahwang16@gmail.com (Y.H.); bluedogy77@gmail.com (I.P.); haydencl08@yahoo.com (H.L.); jennapark7090@gmail.com (J.P.); 5 Comagine Health, Las Vegas, NV 89118, USA; jreeves@comagine.org; 6 Department of Medicine, William Bee Ririe Rural Health Hospital and Clinic, Ely, NV 89301, USA; 7 Department of Medicine, Veterans Affairs Southern Nevada Health Care, North Las Vegas, NV 89086, USA; 8 Geriatric Education Center, Veterans Affairs Southern Nevada Health Care, North Las Vegas, NV 89086, USA; 9 School of Dental Medicine, University of Nevada, Las Vegas, NV 89154, USA; mary.tabrizi@unlv.edu; 10 Department of Family Medicine, Severance Hospital, Yonsei University College of Medicine, Seoul 03722, Republic of Korea; familydoctor@yuhs.ac; 11 Department of Family Medicine, College of Medicine, Chungbuk National University Hospital, Cheongju 28644, Republic of Korea; shrimp611@cbnu.ac.kr; 12 Department of Family Medicine, Yonsei University Wonju College of Medicine, Wonju 26426, Republic of Korea; medeus115@yonsei.ac.kr

**Keywords:** Alzheimer’s, cost-effectiveness, distribution, minorities, primary care, race, telehealth

## Abstract

To people living with Alzheimer’s Disease-Related Disorders (ADRD), timely and coordinated communication is essential between their informal caregivers and healthcare providers. In provider shortage areas, for example, the state of Nevada, telehealth can be an effective primary care delivery alternative to in-person visits. To evaluate the cost-effectiveness of telehealth visits for people living with ADRD in the state of Nevada, a decision-analytic Markov model was developed from healthcare system perspectives with a 10-year horizon/1-year cycle. To estimate the effects of demographic and geographic parameters on the Markov model, race parameters were divided into non-Hispanic White individuals vs. others and location parameters were divided into urban vs. rural. A 12-item short-version Zarit Burden Interview (ZBI-12) was applied to measure the informal caregiver burdens of non-institutionalized people living with ADRD. The values of mortality rate and healthcare utilization were obtained from healthcare systems’ publicly available payor administrative data and Nevada State Inpatient/Emergency Department datasets. Among urban-residing non-Hispanic White individuals, the Incremental Cost-Effectiveness Ratio (ICER) per modified ZBI-12 indicated a cost saving of USD 9.44 with telehealth visits; among urban-residing racial minorities, the ICER per modified ZBI-12 indicated a cost saving of USD 29.26 with in-person visits; and among rural residents, the ICER per modified ZBI-12 indicated a cost-saving of USD 320.93 with telehealth visits. Distributional differences in the cost-saving effects of telehealth primary care were noted in line with racial and geographic parameters. Workforce and caregiver training is necessary for reducing distributional differences, especially among urban-residing racial monitories living with ADRD in the provider shortage area of the state of Nevada.

## 1. Introduction

To people living with Alzheimer’s Disease-Related Disorders (ADRD), timely and coordinated communication is essential between their informal caregivers and healthcare providers [1]. Specifically, communication between caregivers and primary care providers determines the quality and efficiency of care for people living with ADRD [2]. The state of Nevada is a provider shortage state, ranking as having the fifth fewest primary care providers per 100,000 capita among the 50 states (192.6 vs. 232.0 U.S. average [3]). As a result, Nevada ranks highest in terms of 30-day hospital readmission rates (25.8%); it is the third highest ranking state in terms of ADRD care expenditure due to ED visits and hospital readmissions for Medicare beneficiaries [4]. The COVID-19 pandemic led to the challenges of transportation and social isolation for people living with ADRD and their caregivers. In response, telehealth utilization has been facilitated with legal and financial support; for example, the CARES Act and American Rescue Plan was initiated. In response, the state of Nevada established a resources and education network—Nevada COVID-19 Aging Network Rapid Response (Nevada CAN)—operated by Nevada’s Aging Disability Services Division [5]. Two academic geriatric institutions (University of Nevada, Reno Sanford Center for Aging and University of Nevada, Las Vegas) and a quality improvement organization (Comagine Health) led geriatric workforce and informal caregiver telehealth training for people living with ADRD in the state of Nevada. As a result, geriatric workforce and informal caregiver telehealth training for people living with ADRD in the state of Nevada resulted in improvements in advance care planning, but the challenge of health disparities among racial minorities residing in urban areas was noted [2,6].

The top 5% of healthcare utilizations for complex medical conditions, for example, ADRD, account for 60% of the healthcare expenditure in the U.S. [7,8]. Because concentrated healthcare expenditures are major public burdens, telehealth as an alternative primary care delivery method might save time and reduce the effort of transportation, as well as prevent the labor productivity loss of caregivers for people living with ADRD in the provider shortage area of the state of Nevada. The aim of this study is to compare the cost-effectiveness of primary care delivery in person vs. telehealth visits for people living with ADRD in the state of Nevada.

## 2. Materials and Methods

### 2.1. Overview

**Figure 1:**
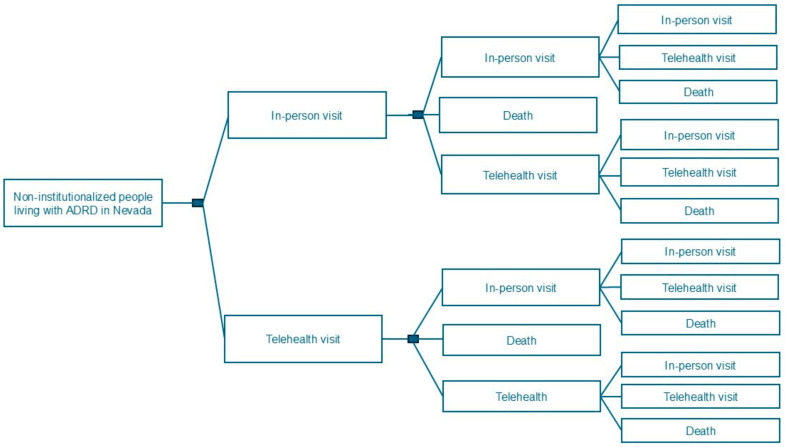
Markov model of choosing primary care delivery tools: In-person vs. telehealth visits.

The Institutional Review Board (IRB) at the University of Nevada, Las Vegas (UNLV), exempted review of this study because it was determined to be a quality improvement/evaluation. Therefore, this study was not subject to IRB approval and oversight evaluation as human subject research (#1613064-1 and 2014-18). We developed a Markov model that simulated the choice of primary care delivery tools: in-person vs. telehealth visits for non-institutionalized people with ADRD. The structure of the model is shown in Figure 1.

### 2.2. Study Cohort

**Figure 2:**
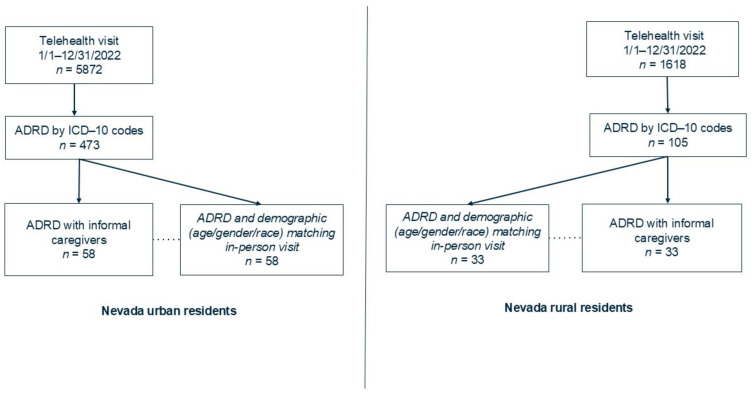
Study cohort selection process of Nevada urban and rural residents.

The base-case population comprised community-dwelling people living with ADRD in the state of Nevada of the U.S. From the electronic health record (EHR) and claim data of urban and rural health systems, 5872 and 1168 primary care telehealth visits were identified, respectively. Using the International Classification of Disease, Tenth Revision, Clinical Modification (ICD-10-CM) ADRD diagnostic codes of either F01, F02, or F03, 473 and 105 ADRD telehealth visits were identified from urban and rural health systems, respectively. Finally, we defined telehealth visit users’ individual profiles, finding 58 urban and 33 rural Nevada residents with available informal caregivers. Informal caregivers were unpaid for their caregiver role and lived in the same geographic area of the individual living with ADRD. They were identified from the EHR administrative data. To match demographic characteristics (age, gender, race) with in-person visit users, the multivariate data matching (MDM) process was applied to data extracted from the EHRs of urban and rural health systems [9]. Sample size was determined by a confidence level of 85% and a margin of error within 10% for urban residents, and a confidence level of 75% and a margin error within 10% for rural residents. Figure 2 presents the process of establishing the study cohort of Nevada urban and rural residents. STATA, version 17 (Stata Corp, College Station, TX, USA), was used for this matching process and sample size determination. A single payment program (Medicare Advantage) administrative and utilization claim data source was used in urban and rural health systems from 1 January 2022 to 31 December 2022.

### 2.3. Parameters

We used data on transition probabilities and mortality rate from urban and rural health systems. Both urban and rural health systems are safety-net and critical-access organizations in the state of Nevada. The model was developed from a third-party payer perspective. All costs were converted to a 2022 baseline using the healthcare inflation rate published elsewhere [10]. Because of the assumption of the same cost between in-person and telehealth primary care visits for people living with ADRD, we estimated costs of (a) telehealth training personnel labor costs for telehealth visits and (b) traveling and labor productivity loss costs for in-person visits. Using this study’s internal personnel labor cost as of fiscal year 2022, telehealth training personnel labor cost was calculated from trainer’s hourly wages and the conversion probability from in-person visits to telehealth visits was calculating using urban and rural health system data. Traveling cost was estimated from the distance between the given home address and healthcare provider address. Labor productivity loss was estimated by the difference between the roundtrip time required to travel from the home address to an in-person primary care clinic, plus in-person primary care visit time vs. the time required to attend a telehealth visit from home (i.e., time savings = roundtrip drive time + [time for in-person primary care visit − time for telehealth visit]) [11]. In addition, we estimated emergency department (ED) visits and the hospitalization-associated costs of in-person and telehealth visits. ED visit and hospitalization-associated utilization information was collected from the Medicare Advantage payor programs. We also verified this information by reviewing ED/hospital discharge summaries, including principal diagnoses and hospital length-of-stay information from EHRs. The ED-visit-associated cost was estimated from the Nevada State Emergency Department Database (SEDD), and hospitalization-associated cost was estimated from the Nevada State Inpatient Database (SID), which is a publicly available dataset, for the period between 1 January 2021 and 31 December 2021 [12,13]. The Nevada SEDD and SID contain the ED visit and hospital discharge records of all community hospitals in the state of Nevada, and were originally developed for the Healthcare Cost and Utilization Project (HCUP) by Agency Healthcare Research and Quality (AHRQ). The Nevada SEDD and SID cover more than 95% of all Nevada ED visits and hospital discharges [12,13]. The Nevada SEDD and SID include anonymous patient-level information, including demographics, diagnostic/procedure codes, ED visits, and hospital utilizations. We collected the number of ED visits and hospitalizations at an individual level, as well as lengths of stay (days) and hospital charges per principal diagnoses from the Nevada SEDD and SID. Then, ED-visit-associated and hospitalization-associated costs were estimated by combining the hospital lengths of stay (days) and the daily average hospital charges per principal diagnoses [2]. ED-visit-associated and hospitalization-associated cost estimates were weighted by age and gender [2]. We assigned caregiver burden as a substitute for quality-of-life (QoL) weights because the direct QOL assessment of people living with ADRD is often challenging to validate, especially for those with severe/advanced ADRD [14]. Caregivers are decision-makers on actual healthcare utilization, for example, calling 911 to go to the ED on behalf of a person living with ADRD. The short-form 12-item Zarit Burden Interview (ZBI-12) has been validated to quantify the burden of caregivers for people living with ADRD [15]. Possible ZBI-12 scores range from 0 (minimum burden) to 48 (maximum burden). We modified a scoring matrix with a range from 0 (maximum burden) to 100 (minimum burden). This study used the Consolidated Health Economic Evaluation Reporting Standards (CHEERS) reporting guideline [16].

### 2.4. Cost-Effectiveness Analysis

To estimate the effects of demographic and geographic parameters on the Markov model, race parameters were divided into non-Hispanic White individuals vs. others, and location parameters were divided into urban vs. rural. We divided scenarios into the following three models: (A) urban-residing non-Hispanic White individuals, (B) urban-residing racial minorities, and (C) rural residents. We estimated the Incremental Cost-Effectiveness Ratio (ICER) of telehealth visits. Because of the limited life expectancy of people living with ADRD, we used a 10-year horizon with a yearly cycle length to advance time in the model. We discounted all future costs and ZBI-12 at 3% per year. All Markov models were constructed using TreeAge Pro Healthcare version 2024 R1.1 (TreeAge Software Inc., Williamstown, MA, USA) to assess the ICERs of telehealth visits in all three Markov models.

## 3. Results

As presented in Table 1, of the 58 Nevada urban residents, the mean age was 81.3 years, with a standard deviation of 9.7 years and a range of 61–98 years. Approximately one-third (37.9%) was 79 years and younger. In terms of gender, 41.4% were male, 55.3% were female, and 3.6% were other. More than half (51.7%) of telehealth visits were to non-Hispanic White individuals; 15.5% were to non-Hispanic Black individuals; 13.8% were to Hispanic or Latino individuals; 12.1% were to non-Hispanic Asian individuals; and 6.9% were to other or mixed-race individuals. Of the 33 Nevada rural residents, the mean age was 82.6 years, with a standard deviation of 6.7 years and a range of 63–89 years. Approximately half (45.4%) were 79 years and younger. In terms of gender, 39.4% were male and 60.6% were female. More than three-fourths (84.8%) of telehealth visits were to non-Hispanic White individuals; 3.0% were to non-Hispanic Black individuals; 6.1% were to Hispanic or Latino individuals; and 6.1% were to other or mixed-race individuals.

Table 2 presents three Markov models and the analysis results by demographic and geographic parameters.

In Model A, concerning urban-residing non-Hispanic White individuals, the probability per year of transitioning from telehealth visits to in-person visits (18.29%) was lower than that from in-person visits to telehealth visits (31.37%). The mortality rate per year of telehealth visit users (8.10%) was lower than that of in-person visit users (11.47%). The mean numbers of ED visits and hospitalization per year in telehealth visit users (1.52, 0.74) were lower than those of utilizing in-person visits (2.24, 1.86), respectively. The incremental cost of telehealth visits (USD 202) was lower than that of in-person visits (USD 230). The ICER per modified ZBI-12 for telehealth visits was USD 9.44 lower than that of in-person visits. In Model B, concerning urban-residing racial minorities, the probability per year of transitioning from telehealth visits to in-person visits (26.77%) was higher than that from in-person visits to telehealth visits (21.67%). The mortality rate of telehealth visit users per year (11.25%) was lower than that of in-person visit users (16.90%). The mean numbers of ED visits and hospitalization per year in telehealth visit users (1.52, 1.22) were lower than those in-person visit users (2.24, 2.71), respectively. The incremental cost of telehealth visits (USD 220) was lower than that of in-person visits (USD 237). The ICER per modified ZBI-12 for telehealth visits was USD 29.26 higher than that of in-person visits. In Model C, concerning rural residents, the probability per year of transitioning from telehealth visits to in-person visits (30.00%) was higher than that from in-person visits to telehealth visits (26.67%). The mortality rate of telehealth visits users per year (7.52%) was lower than that of in-person visit users (10.29%). The mean numbers of ED visits and hospitalization per year for telehealth visit users (0.93, 0.61) were lower than those of in-person visit users (1.20, 0.96), respectively. The incremental cost of telehealth visits (USD 436) was lower than that of in-person visits (USD 503). The ICER per modified ZBI-12 for telehealth visits was USD 320.93 lower than that of in-person visits.

## 4. Discussion

The purpose of this study is to estimate the cost-effectiveness of telehealth visits as an alternative to in-person visits for people living with ADRD in the state of Nevada, a provider shortage state. The main findings are distributional differences in the cost-saving effects of telehealth primary care in line with racial and geographic parameters. Among urban-residing non-Hispanic White individuals, the ICER per modified ZBI-12 indicates a USD 9.44 cost-saving with telehealth visits; among rural residents, the ICER per modified ZBI-12 indicates a USD 320.93 cost-saving with telehealth visits. Although this study used healthcare perspectives, our Markov models were divided into three models by racial and geographic parameters based on the health equity considerations of this study [17]. These findings align with the hypothesis that the benefits of telehealth visits may facilitate timely and better communication for people living with ADRD than in-person visits. The cost-saving effect of the telehealth visits was the result of reduced ED visits and hospitalizations across the Markov models. This reduced utilization of healthcare led to the enhancement of care efficiency, as noted in the previous literature on the benefits of telehealth for people living with ADRD [1,2]. In the midst of the payment system transition of Medicare beneficiaries from traditional fee-for-service payments to bundle payments, this study’s findings (i.e., the cost-saving effects of telehealth as a result of reducing ED visits/hospitalizations) give an insight into how to reform healthcare provider payments to achieve greater efficiency and value in providing primary care to people living with ADRD [18]. As the Medicare Advantage program was the single payer of this study, telehealth’s cost-saving effects have implications for advancing primary care efficiency for the population of rural residents in this study.

On the other hand, among urban-residing racial minorities, the ICER per modified ZBI-12 indicates a USD 29.26 cost-saving with in-person visits compared to telehealth visits. Although healthcare utilizations in telehealth visit users were fewer than those in in-person visit users among urban-residing racial minorities, a higher transition probability from telehealth visits to in-person visits (26.77%) was noted compared to that from in-person visits to telehealth visits (21.67%). These findings were the opposite to those found for delivery-type transition probability among urban-residing non-Hispanic White individuals (18.29% from telehealth visits to in-person visits; 31.37% from in-person visits to telehealth visits). These findings are consistent with previous telehealth utilization studies that identified that racial minorities living with ADRD were less likely persist with telehealth visits compared to non-Hispanic White individuals living with ADRD [6,19]. Using the same dataset, the previous study examining the racial disparities of telehealth recipients identified that this disparity may have been exacerbated by discussing mentation during telehealth visits [6]. Similar findings and interpretations were noted in another telehealth disparity study in the northeastern area [20]. To reduce disparities related to cost burdens in telehealth visits for urban-residing racial minorities, workforce training is a realistic priority strategy. The Age-Friendly Health System 4Ms framework (What Matters, Mobility, Medication, Mentation) is a locally and interprofessionally adoptable training toolkit for the workforce caring for people living with ADRD, from entry level to leadership [21,22]. This 4Ms framework is also optimal for training the informal caregivers of people living with ADRD to enhance their capacity to cope with the challenges of caring for ADRD individuals via the authentic care partnership model [23].

Finally, as telehealth has been adopted as a modality of care for people living with ADRD since the COVID-19 pandemic, it is essential to develop and support user-friendly health information technology for care partners [1,2]. This is crucial for timely communication between caregivers and healthcare providers in transitional care coordination, especially within two weeks of discharge from acute hospital or/and skilled nursing facilities [1,2]. In provider shortage areas such as the state of Nevada, telehealth can play a pivotal role in identifying community-dwelling individuals with ADRD and referring them to appropriate resources at an early stage. Evidence-based training for the workforce and for caregivers for people living with ADRD is essential to sustain telehealth as a primary care visit delivery tool, especially among racial minorities. If the State Legislature in the 2025 session approves statewide efforts of this initiative, established partnerships among Nevada ADRD stakeholders (University of Nevada, Reno Sanford Center for Aging, Nevada State Aging Disability Services Division, Alzheimer’s Association, Cleveland Clinic Lou Ruvo Center for Brain Health, and Kirk Kerkorian School of Medicine at UNLV) can augment the benefits of telehealth visits’ cost-saving effects for community-dwelling Nevadans living with ADRD.

Telehealth primary care delivery has environmental output implications in all sectors of the economy, both in the short and long term [24]. The interactions between per capita health expenditures and per capita atmospheric pollution have been empirically examined elsewhere using the well-known Environmental Kuznets Curve [25]. Thus, telehealth is a promising method to achieve a greater efficiency and equity of primary care for people living with ADRD and their caregivers in provider shortage areas.

The findings of our study are limited by a single urban and single rural health system with the distinction of safety-net/critical-access systems and a single year of observation, with a single Medicare Advantage payer program.

Further studies with diverse healthcare system partners with different characteristics may generate different findings from our study results. As we reviewed administrative data templates without personal information identification, we did not review the healthcare providers’ progress notes in the EHRs. Therefore, the severity of ADRD was not captured. Although the reasons associated with choosing telehealth or in-person visits were not included in this study, Medicaid and dual Medicare/Medicaid eligible program enrollees were not included because they might have been restricted to only telehealth service reimbursements with audio-only and asynchronous services [26]. Although the majority of telehealth waivers enacted during the COVID-19 public health emergency are set to expire on 31 December 2024, in the absence of legislative action, the CMS has proposed to leave certain key flexibilities in place, including the allowance for physicians and other practitioners to furnish remote “direct supervision” through their immediate availability via audio–video technology [27]. The current CMS reimbursement policy for telehealth may impact the originating site and service site flexibilities from early 2025 [27]. Selection bias might have occurred in the cohort selection process of identifying individuals living with ADRD and their available informal caregivers due to the reliance on data accuracy and administrative data templates. Lastly, we assigned caregiver burden as a substitute for QoL weights. QoL weights are assigned to a particular health state and represent the utility of that state as perceived by society [28,29]. The challenges in the quality-adjusted life years (QALYs) traditional metric for CEA within value assessments might lead to distributive justice being overlooked, resulting in negative consequences for older, sicker stakeholders in communities like people living with ADRD [28,29]. ADRD have a progressive nature. Even with an intervention, telehealth services might not be able to reverse the course of disease progression of people living with ADRD. For example, a 2005 decision using a QALY-based cost-effective assessment from the U.K.’s National Institute of Health and Care Excellence resulted in the denial of cholinesterase inhibitor drugs for ADRD treatment under the U.K.’s National Health Service. As a result, the consumer advocates argued that NICE’s QALY cost-effectiveness assessment neglected to consider caregivers’ benefits from the ADRD treatment [30]. Telehealth primary care can have productivity benefits, enabling the caregivers of people living with ADRD to stay in the formal workforce, perform productive work in their household, volunteer in their community, or provide community resources of value to social welfare. Since the implementation of the Affordable Care Act in the U.S., QALY has been used with substantial limitations due to the Center for Medicare and Medicaid Services barring the QALY criterion to determine the coverage of public insurance [31]. Looking ahead to advance equity perspectives, caregiver burden is a new tool for assessing caregiver burden and healthcare utilizations, ED visits, and hospitalizations.

## 5. Conclusions

Among urban-residing non-Hispanic White individuals, the ICER per modified ZBI-12 indicates a USD 9.44 cost-saving with telehealth visits; among urban-residing racial minorities, the ICER per modified ZBI-12 indicates a USD 29.26 cost-saving with in-person visits; among rural residents, the ICER per modified ZBI-12 indicates a USD 320.93 cost-saving with telehealth visits. Distributional differences in the cost-saving effects of telehealth primary care were noted in line with racial and geographic parameters. Workforce and caregiver training is necessary for reducing distributional differences, especially among urban-residing racial monitories living with ADRD in provider shortage areas such as the state of Nevada.

## Figures and Tables

**Table 1:** Cohort demographic and geographic description.

		Nevada Urban Residents, *n* = 58		Nevada Rural Residents, *n* = 33	
		*n*	%	*n*	%
Age	Mean ± standard deviation (range)	81.3 ± 9.7 (61~89)		82.6 ± 6.5 (63~89)	
	~79	22	37.9	15	45.4
	80~	36	62.1	18	54.6
Gender	Male	24	41.4	13	39.4
	Female	32	55.3	20	60.6
	Other	2	3.6	0	0
Race	Non-Hispanic White	30	51.7	28	84.8
	Non-Hispanic Black	9	15.5	1	3.0
	Hispanic, Latino	8	13.8	2	6.1
	Non-Hispanic Asian	7	12.1	0	0
	Other or mixed race	4	6.9	2	6.1

**Table 2:** Markov models and analysis results by demographic and geographic parameters.

Model A. Urban-Residing Non-Hispanic Whites							
			**Event Number/Year** **Mean**				
**Delivery Type**	**Transition to Other Delivery Probability %/Year**	**Mortality Rate** **%/Year**	**ED Visit** **USD 6177/Event**	**Hospitalization** **USD 34,950/Event**	**Incremental Cost** **USD, Mean**	**Modified ZBI-12** **(Total 0–100), Mean**	**ICER/Modified ZBI-12** **USD**
In-person visit	From in-person to telehealth visit 31.37	11.47	2.24	1.86	230	70.28	Ref.
Telehealth visit	From telehealth to in-person visit 18.29	8.10	1.52	0.74	202	67.35	−9.44
Model B. Urban-Residing Racial Minorities							
			**Event Number/Year** **Mean**				
**Delivery Type**	**Transition to Other Delivery Probability %/year**	**Mortality Rate** **%/Year**	**ED Visit** **USD 6459/Event**	**Hospitalization** **USD 40,635/Event**	**Incremental Cost** **USD, Mean**	**Modified ZBI-12** **(Total 0–100), Mean**	**ICER/Modified ZBI-12** **USD**
In-person visit	From in-person to telehealth visit 21.67	16.90	2.24	2.71	237	72.94	Ref.
Telehealth visit	From telehealth to in-person visit 26.77	11.25	1.52	1.22	220	73.55	29.26
Model C. Rural Residents							
			**Event Number/Year** **Mean**				
**Delivery Type**	**Transition to Other Delivery Probability %/Year**	**Mortality Rate** **%/Year**	**ED Visit** **USD 5193/Event**	**Hospitalization** **USD 30,346/Event**	**Incremental Cost** **USD, Mean**	**Modified ZBI-12** **(Total 0–100), Mean**	**ICER/Modified ZBI-12** **USD**
In-person visit	From in-person to telehealth visit 26.67	10.29	1.20	0.96	503	60.58	Ref.
Telehealth visit	From telehealth to in-person visit 30.00	7.52	0.93	0.61	436	60.37	−320.93

## Data Availability

The data presented in this study are available on request from the corresponding author. The data are not publicly available due to privacy or ethical restrictions.
